# Comparison Study of Multiple Machine Learning Models for Predicting Anxiety Among Neurosurgical ICU Family Members Using Critical Care Family Needs Inventory

**DOI:** 10.1155/jonm/1384539

**Published:** 2026-07-29

**Authors:** Feng Zhang, Cunyi Huo, Ping Xu, Ruixiang Sun, Huayue Zhang, Xiaoxue Liu, Shengxiang Zhang, Lei He, Huifen Kuai

**Affiliations:** ^1^ Department of Neurosurgery, The First Affiliated Hospital of Wannan Medical University (Yijishan Hospital of Wannan Medical University), Zheshan West Road on the 2nd, Wuhu 241000, Anhui, China; ^2^ Department of Cardiology, The First People’s Hospital of Chuzhou City, No. 369 Zuiwang West Road Nanqiao District, Chuzhou 239001, Anhui, China; ^3^ Department of Cardiology, The First Affiliated Hospital of Soochow University, No. 899 Pinghai Road, Suzhou 215000, Jiangsu, China, sdfyy.cn

**Keywords:** anxiety status, Critical Care Family Needs Inventory (CCFNI), family needs, machine learning, neurosurgical intensive care unit (NICU), precision nursing

## Abstract

**Aim:**

This investigation intended to develop machine learning predictive models to predict anxiety status of neurosurgical ICU patients’ relatives by taking all Critical Care Family Needs Inventory (CCFNI) items as predictive variables, identify core unmet family demands strongly correlated with psychological distress, and select the optimal model for clinical rapid anxiety screening to support targeted precise nursing interventions.

**Methods:**

A total of 1000 first‐degree relatives of neurosurgical ICU patients with a Glasgow Coma Scale (GCS) score ≤ 8 at the First Affiliated Hospital of Wannan Medical University from January 2024 to December 2025 were enrolled as research subjects. On the third day after admission to the department, general clinical data, HADS scores, and CCFNI scale data of the subjects were collected. SPSS 26.0 was applied for traditional statistical analysis and R 4.3.3 was utilized to develop machine learning prediction models. The full dataset was randomly split into training and test sets at a 7:3 ratio. Multiple metrics including accuracy, sensitivity, specificity, F1‐score, and AUC were adopted to thoroughly assess model efficacy, followed by screening core predictors linked to relatives’ anxiety levels. The research obtained ethical clearance from our institutional review board (ID: 2018043) and fully abided by the ethical standards outlined in the Declaration of Helsinki.

**Results:**

Among 1000 neurosurgical ICU patients, the primary diagnoses were craniocerebral injury (40.2%) and hypertensive intracerebral hemorrhage (39.6%) constituted the primary diagnoses, and the majority of patients served as the main economic source of their families (60.4%). The average age of family members was 51.72 ± 15.22 years, with junior and senior high school education being the predominant educational attainment, and nearly half of them had no accompanying experience. Screening of family members’ anxiety status showed that only 1.2% had no anxiety symptoms, and the incidence of moderate to severe anxiety was as high as 76.8%. Model validation was performed using a 7:3 ratio and leveraged 10‐fold cross‐validation to optimize hyperparameters. The comparison results of three machine learning models indicated that the support vector machine (SVM) algorithm delivered superior overall predictive efficacy, achieving favorable accuracy (0.8863), sensitivity (0.7264), specificity (0.9348), and F1 score (0.7463), and its AUC value reached 0.9364; the Random Forest model had the highest AUC value (0.9451) but relatively low sensitivity (0.5362). The Artificial Neural Network exhibited weaker overall predictive capacity relative to the other two algorithms. Variable importance analysis of the SVM model revealed that CCFNI7, CCFNI12, and CCFNI9 were the core indicators for predicting family members’ anxiety status.

**Conclusion:**

The incidence of moderate to severe anxiety among neurosurgical ICU patients’ relatives far exceeds that of patients’ families in general ICU and anxiety symptoms are closely correlated with neurosurgical ICU specific stressors and unmet family demands. Compared with traditional statistical methods, machine learning algorithms exhibit prominent strengths in predicting family anxiety and screening core CCFNI items related to psychological distress. The SVM model can serve as a reliable clinical screening tool, which may support medical–family collaborative precise nursing and help relieve anxiety symptoms among relatives.

**Implications for Practice:**

The optimal machine learning model screened in this study can assist clinicians in precisely recognizing the core demands of neurosurgical ICU patients’ relatives, which offers evidence to develop personalized intervention protocols, elevating the standard of humanistic intensive care nursing and optimizing the medical experience of both patients and their relatives.

**Reporting Method:**

This study strictly followed the TRIPOD statement for transparent reporting of diagnostic and prognostic prediction models, which provides standardized reporting checklists for all model development and validation procedures [1]. Meanwhile, we referenced the integrated risk factor analysis framework proposed by Wang et al. to standardize the methodological interpretation of multidimensional predictive indicators in this research [2].

**Patient or Public Contribution:**

Family members of Neurosurgical ICU patients cooperated in completing relevant questionnaires, providing core data support for the study. There was no patient or public contribution.

**Implication for Nursing Management:**

The proposed model empowers nursing administrators to streamline family need assessment, optimize manpower allocation, and standardize emotional intervention procedures. It facilitates refined Neurosurgical ICU nursing management and improves holistic nursing quality.

## 1. Introduction

As the primary clinical ward dedicated to severe patient monitoring, the intensive care unit (ICU) often places patients’ relatives under various stressors that impair their physical, mental, and social health, with this burden far more prominent in Neurosurgical ICU. The Neurosurgical ICU admits patients with high risks of neurological impairment, unpredictable disease progression, and strong dependence on medical treatment. Such patients usually suffer life‐threatening and volatile conditions. Research reveals that severe traumatic brain injury patients hospitalized in neurosurgical ICUs carry an in‐hospital mortality rate up to 46%, with a 40% rate of functional impairment observed at the 6‐month postoperative follow‐up [3, 4]. As continuous emotional supporters throughout the treatment process, they undergo psychological changes that correspond with the clinical conditions of patients. Persistent anxiety damages caregivers’ physical and mental well‐being, and such negative emotions may also impede patients’ postoperative rehabilitation via emotional contagion—an issue frequently underestimated by healthcare professionals [5].

Research studies focusing on psychological conditions of NICU patient relatives are still scarce; however, with the annual increase in the number of Neurosurgical ICU admissions, it has become an increasingly important issue in the field of public health. As a globally acknowledged scale, the Critical Care Family Needs Inventory (CCFNI) is applied to assess the practical and emotional needs of families caring for critically ill individuals, which serves as a vital link between healthcare provision and the actual needs of families [6].

We classified core demands of patient relatives into several dimensions covering information access and emotional support, care participation, and environmental safety; the scale provides clinicians with accurate data for developing targeted intervention strategies. Currently, analyses of CCFNI data in clinical research mainly have primarily adopted conventional statistical methods. The strength of these methods lies in their logical rigor, allowing verification of correlations between family needs and related variables. However, conventional statistical methods have notable limitations—they are unable to identify latent patterns within needs assessments or uncover deeper unmet needs embedded in the data. The emergence of machine learning has introduced a new approach for advanced CCFNI analysis. Compared with conventional statistical techniques, machine learning demonstrates greater adaptability to complex data structures; it can capture intricate interactions among multidimensional CCFNI variables, predict family anxiety, and screen core unmet demand items strongly associated with psychological distress among high‐risk relatives [7]. A study by Seren et al., which focused solely on the decision‐making preferences of relatives of Neurosurgical ICU patients, found that approximately 61% preferred joint decision‐making, whereas 12% favored a passive approach [8]. Dupont et al. applied machine learning methods to predict PTSD in ICU patients’ families and achieved good results [9]. However, existing machine learning studies related to ICU family psychological outcomes rarely conduct head‐to‐head comparisons of multiple algorithms specifically based on CCFNI indicators, and few prior works systematically screen the optimal predictive model tailored to CCFNI datasets for Neurosurgical ICU family anxiety prediction.

With the concept of precision medicine becoming increasingly integrated into critical care nursing, accurately identifying family needs reflected by the CCFNI has become central to improving the standard of personalized clinical care [10]. In the Neurosurgical ICU, the requirements of family members are notably heterogeneous; even within the same category of needs, the priority ranking may vary considerably depending on personal experience, cognitive level, and family role. Therefore, accurate CCFNI‐based identification not only enables healthcare professionals to design individualized nursing plans for family members with different need subtypes but also facilitates proactive adjustment of nursing services by predicting changes in needs. Ultimately, this approach enhances the fulfillment of family members’ needs and improves the overall care experience of patients and families, while offering novel technical references and operational suggestions to advance high‐standard construction of critical care nursing services. This research intended to construct various machine learning models to forecast anxiety among family caregivers and identify key CCFNI entries inducing psychological distress and select the optimal model for clinical use, thereby providing a theoretical basis for implementing precise clinical interventions. In this study, anxiety status (Hospital Anxiety and Depression Scale [HADS] > 7) serves as the target variable, while CCFNI items are used as predictors. The CCFNI scale was originally developed to systematically quantify explicit and implicit care‐related needs of ICU family members, and existing scale validation studies confirm its capacity to capture both overt and latent unmet demands of relatives [11]. Guided by the stress process model of family caregiving, unmet care demands can act as chronic stressors that correlate with elevated psychological distress. On this theoretical basis, we hypothesize that unmet family needs measured by the CCFNI are closely linked to elevated anxiety risk; therefore, predicting anxiety from CCFNI items enables the identification of which unmet needs correlate most strongly with psychological distress [12].

## 2. Sample

### 2.1. Clinical Data

A total of 1042 family members of Neurosurgical ICU patients with a Glasgow Coma Scale score ≤ 8 who were admitted between January 2024 and December 2025 were initially recruited from the Department of Neurosurgery, Yijishan Hospital of Wannan Medical University. After strict selection in line with per‐set eligibility standards, 1000 eligible subjects were retained for subsequent statistical analyses. All questionnaires were distributed 3 days following ICU admission of each patient on the third day.

Inclusion criteria were as follows: (1) first‐degree relatives (spouse, parents, or children) of Neurosurgical ICU patients who served as the primary decision‐makers regarding the patients’ care; (2) individuals who accompanied the patients for ≥ 3 days during their ICU stay and were able to complete the independently and cooperatively; and (3) individuals without history of psychiatric disorders.

Exclusion criteria were as follows: (1) family members suffering from severe physical illnesses that could influence their ability to complete the questionnaire; (2) patients who were transferred to general wards within 3 days after Neurosurgical ICU admission; and (3) patients death occurring the survey period. The entire process of participant screening and data collection was conducted by a multidisciplinary team consisting of one attending psychologist, one attending neurosurgeon, and one nurse.

**Figure 1:**
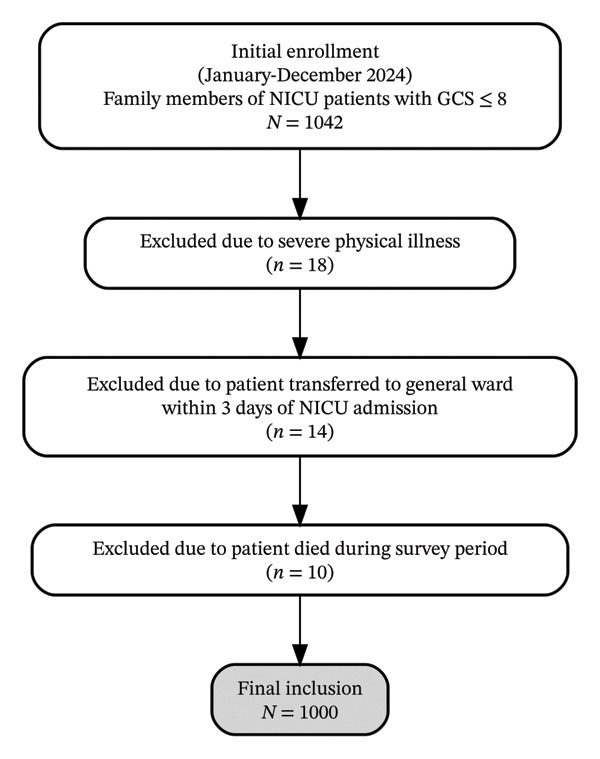
Flowchart showing the recruitment, exclusion, and final allocation of family members of neurosurgical ICU patients.

All experimental procedures complied with relevant ethical norms and institutional regulations and were implemented following the core tenets of the Declaration of Helsinki. This research was formally approved by the Ethics Committee of the First Affiliated Hospital of Wannan Medical University (Ethics No.: 2018043). The procedures for participant recruitment, exclusion, and dataset allocation are shown in Figure 1.

Figure 1 illustrates the recruitment, exclusion, and final allocation of family members of Neurosurgical ICU patients. A total of 1042 potential participants were initially enrolled. After excluding 42 participants due to excessive missing data on questionnaires, a total of 1000 eligible participants were ultimately enrolled for final statistical analysis. The overall dataset was randomly assigned to the training cohort (*n* = 700, 70%) and a test set (*n* = 300, 30%).

### 2.2. Observation Indicators

#### 2.2.1. General Clinical Data

The general clinical data collected included patient’s admission date, diagnosis, and the family member’s gender, age, educational level, ethnicity, religion, relationship to the patient, monthly per capita household income (in yuan), occupation, and prior accompanying experience.

#### 2.2.2. HADS [13]

The HADS quantifies anxious and depressive moods across ordinary residents and various patient cohorts, and it fits quick mental state screening within nonpsychiatric clinical wards. This scale consists of 14 questions split into two separate subdimensions for anxiety and depression, with seven items apiece. Every question adopts a 0–3 scoring range, and the anxiety subscale yields total scores between 0 and 21. Score stratification standards are set as 0–7 points for absent anxiety, 8–10 for mild symptoms, 11–14 for moderate conditions, and 15–21 for severe anxiety, supporting quantitative grading of anxiety levels. Featuring brevity and convenient operation, HADS owns favorable reliability and validity. The HADS is concise and efficient and demonstrates high reliability and validity.

#### 2.2.3. CCFNI

The CCFNI serves as a dedicated measurement tool to identify care demands of ICU patient relatives across intensive care wards. This scale mainly serves to systematically sort out caregivers’ multifaceted demands, such as access to disease‐related information, psychological comfort, and the right to take part in patient care and environmental safety, thereby providing an evidence‐based foundation for developing individualized clinical care plans. The CCFNI boasts stable psychometric properties and favorable cross‐cultural adaptability. This scale can detect overt and hidden demands of patient caregivers and has been extensively adopted in clinical work and relevant studies focused on intensive care relatives.

### 2.3. Data Analysis

This research integrated conventional statistical methods with machine learning architectures to process collected data. SPSS Version 26.0 was used for conventional statistical analyses, and R Version 4.3.3 was employed to construct machine learning models. Normality testing was performed on all continuous variables. Variables conforming to a normal distribution were expressed as mean ± standard deviation ($\overset{¯}{x}$ ± *s*), whereas nonnormally distributed variables were presented as median (interquartile range) [M (Q1, Q3)].

To further explore the structure of family members’ needs and their complex relationship with anxiety, machine learning techniques were incorporated. A feedforward neural network with a multilayer perceptron architecture was developed using the Keras framework to perform deep learning on CCFNI data collected from family members of critically ill patients. Through nonlinear transformations across the input, hidden, and output layers, the model achieved in‐depth mining and pattern recognition of multidimensional demand features. It subsequently performed priority ranking of family members’ core needs, analyzed potential correlation pathways among needs, and clustered the influencing factors associated with family members’ anxiety. The application of this approach aimed to address the inherent drawbacks of conventional linear statistical models in handling high‐dimensional and nonlinear data, thereby enhancing both the accuracy and depth of demand analysis.

## 3. Results

### 3.1. Baseline Characteristics of Neurosurgical ICU Patients and Their Family Members

Baseline profiles of 1000 neurosurgical intensive care patients alongside their kin are summarized in this segment. Subjects had a mean age of 54.92 ± 14.56 years, a slight predominance of males (507 cases, 50.7%). Traumatic brain injury (402 cases, 40.2%) and hypertensive intracerebral hemorrhage (396 cases, 39.6%). Median duration from the emergence of illness symptoms to hospitalization was 12.2 (6–18) hours, and the duration of ICU stay was 10.6 (5.6–15.6) days. Analysis of medical insurance categories showed employee medical insurance as the predominant type, covering 511 participants (51.1%). A majority of the patients were the primary financial providers for their families (604 cases, 60.4%).

The average age of participating relatives was 51.72 ± 15.22 years, with a nearly equal gender distribution. Junior high and senior high school education constituted the two largest educational groups, accounting for 314 (31.4%) and 306 (30.6%) subjects, respectively. Spouses and adult children constituted the primary caregivers, with 404 (40.4%) and 397 (39.7%) participants, respectively. The monthly per capita household income was predominantly within the 3000–5000 yuan range (488 cases, 48.8%), and majority family members were employed (511 cases, 51.1%). Over half of all (533 subjects, 53.3%) had never previously accompanied hospitalized patients (see Table 1).

**TABLE 1:** Analysis of general data of 1000 patients in the neurosurgical intensive care unit (NICU) and their family members.

Indicator	Results
Gender	54.92 ± 14.56
Age	
Male (%)	507 (50.7)
Female (%)	493 (49.3)
Diagnosis	
Traumatic brain injury (%)	402 (40.2)
Hypertensive intracerebral hemorrhage (surgical treatment) (%)	396 (39.6)
Intracranial aneurysm (%)	97 (9.7)
Intracranial tumor (%)	105 (10.5)
Time from onset to hospital admission (h)	12.2 (6, 18)
NICU length of stay (d)	10.6 (5.6, 15.6)
Medical insurance type	
Self‐payment (%)	79 (7.9)
Resident medical insurance (%)	316 (31.6)
Employee medical insurance (%)	511 (51.1)
Third‐party insurance (%)	94 (9.4)
Whether the patient is the main family breadwinner	
Yes (%)	604 (60.4)
No (%)	396 (39.6)
Family member’s gender	
Male (%)	500 (50.0)
Female (%)	500 (50.0)
Family member’s age	51.72 ± 15.22
Family member’s educational level	
Primary school and below (%)	171 (17.1)
Junior high school (%)	314 (31.4)
Senior high school (%)	306 (30.6)
College degree and above (%)	209 (20.9)
Relationship between family member and patient	
Patient’s children (%)	397 (39.7)
Patient’s parent(s) (%)	92 (9.2)
Patient’s spouse (%)	404 (40.4)
Patient’s Sibling(s) (%)	107 (10.7)
Marital status	
Married (%)	702 (70.2)
Unmarried (%)	92 (9.2)
Divorced or widowed (%)	206 (20.6)
Average monthly household income per person	
< 3000 CNY (%)	303 (30.3)
3000–5000 CNY (%)	488 (48.8)
> 5000 CNY (%)	209 (20.9)
Occupation	
Unemployed (%)	104 (10.4)
Employed (%)	511 (51.1)
Farmer/worker (%)	86 (8.6)
Self‐employed (%)	110 (11.0)
Retired (%)	189 (18.9)
Whether having had accompanying experience	
Yes (%)	533 (53.3)
No (%)	467 (46.7)

### 3.2. Assessment of Anxiety Status Using the HADS

The anxiety status of 1000 family members of Neurosurgical ICU patients was evaluated using the HADS, with scores categorized into four levels according to established criteria: 0–7 indicating no anxiety, 8–10 indicating mild anxiety, 11–14 indicating moderate anxiety, and 15–21 indicating severe anxiety. The results revealed that only 12 participants (1.2%) exhibited no anxiety symptoms, 220 (22.0%) had mild anxiety, 394 (39.4%) had moderate anxiety, and 374 (37.4%) had severe anxiety. Overall, the incidence of moderate‐to‐severe anxiety reached 76.8% (768 cases). Such data demonstrate that psychological distress is widely observed among family members of Neurosurgical ICU patients (see Table 2).

**TABLE 2:** Anxiety status of family members of 1000 NICU patients.

Clinical stratification of anxiety	Result
No symptoms (0–7 points)	12 (1.2)
Mild (8–10 points)	220 (22.0)
Moderate (11–14 points)	394 (39.4)
Severe (15–21 points)	374 (37.4)
Anxiety status (presence/absence)	
Yes (%)	768 (76.8)
No (%)	232 (23.2)

### 3.3. Comparison of Anxiety State Prediction Models Constructed Using Three Machine Learning Algorithms

Three classic machine learning algorithms were adopted in work to construct predictive models for anxiety status—Random Forest (RF), support vector machine (SVM), and Artificial Neural Network (ANN)—to identify the key factors influencing anxiety status and determine the optimal prediction model (see Table 3).

**TABLE 3:** Comparison of performance metrics among the three machine learning models.

Model	Accuracy^∗^	Sensitivity^∗^	Specificity^∗^	Precision^∗^	F1^∗^	AUC
Random Forest	0.8595 (0.8227–0.8997)	0.5362 (0.4308–0.6479)	0.9565 (0.9298–0.9825)	0.7872 (0.7084–0.8524)	0.6379 (0.5864–0.6923)	0.9451 (0.9175–0.9459)
Support Vector Machine	0.8863 (0.8495–0.9231)	0.7264 (0.6230–0.8290)	0.9348 (0.9021–0.9662)	0.7692 (0.6954–0.8426))	0.7463 (0.7162–0.7864))	0.9364 (0.9056–0.9369)
Artificial Neural Network	0.8294 (0.7826–0.8696)	0.6812 (0.5806–0.7911)	0.8739 (0.8267–0.9163)	0.6184 (0.6024–0.6356)	0.6483 (0.6259–0.6705)	0.8916 (0.8487–0.8928)

^∗^Accuracy, sensitivity, specificity, precision, and F1 scores were reported with 95% confidence intervals derived from the bootstrap method with 200 resamples.

### 3.4. Conceptual Framework for Outcome Definition

The primary outcome of the machine learning models was the anxiety status, as defined in the Data Preprocessing section. Anxiety was used as a surrogate outcome for identifying clinically significant unmet family needs because prior research consistently shows that anxiety among ICU family members is not a primary emotional disturbance but rather a downstream consequence of specific unmet needs measured by the CCFNI. Thus, predicting anxiety from CCFNI items enables the identification of which unmet needs are most strongly associated with psychological distress.

### 3.5. Sample Size Justification

The sample size was determined in accordance with the events per variable (EPV) principle. With 1000 participants and 15 predictor variables (CCFNI items), the EPV for the anxiety group (*n* = 768) was approximately 66, which exceeds the recommended minimum of 10–20 for developing stable prediction models. Thus, the available sample size was adequate for model development and internal validation.

### 3.6. Data Preprocessing

Anxiety status was designated as the outcome variable for model construction. Family members with a HADS anxiety subscale score greater than 7 were classified as the anxiety group, while those attaining scores ≤ 7 were grouped as nonanxious subjects. CCFNI1–CCFNI15 were selected as independent predictive variables.

Missing data management: Missing data were handled using listwise deletion for participants with ≥ 20% missing responses on HADS or CCFNI items (42 participants excluded). No imputation was performed.

Outlier handling: Winsorization at the 1st and 99th percentiles was performed for all continuous variables to mitigate the impact of outliers; no outliers were removed.

Feature scaling: All continuous variables were converted into zero‐mean unit‐variance data to remove interference from inconsistent measurement scales.

Quality control: Quality control measures included double data entry and range checks for all variables.

Class imbalance handling: To address class imbalance (768 anxious vs. 232 nonanxious), Synthetic Minority Oversampling Technique (SMOTE) was applied to the training set only, with *k* = 5 nearest neighbors and a sampling rate of 1:1 [14].

The entire dataset underwent random partitioning into training and testing cohorts at a 7:3 split, with consistent anxiety status distribution maintained in both subgroups.

### 3.7. Model Construction and Parameter Settings

General approach: All models were developed using R Version 4.3.3. Grid search coupled with 10‐fold cross‐validation on the training cohort was adopted for hyperparameter optimization. Model optimization was guided by maximizing cross‐validated AUC. Optimal hyperparameters were applied to refit the final predictive model with training data.

RF model: This study adopted an ensemble learning strategy containing 500 decision trees. At each node split, the model randomly selected feature quantities equivalent to the square root of the total feature number. Two complementary quantification standards for variable contribution were utilized to rank feature importance, namely, the mean reduction in prediction accuracy and the mean drop in Gini coefficient. For the SVM model, the radial basis function (RBF) kernel was chosen as the kernel. The penalty parameter and kernel coefficient were optimized using a grid search to enhance the model’s fitting and generalization capabilities. Class probability outputs were generated by enabling the probability estimation function, facilitating subsequent risk stratification analyses. ANNs were constructed a feedforward neural network containing one hidden layer. Cross‐validation helped identify the optimal count of hidden neurons to eliminate under fitting and overfitting. Model weight tuning was realized via back propagation, the sigmoid curve acted to serve as the activation operator, and dynamic learning rate modification was executed in training to raise convergence efficiency.

### 3.8. Model Evaluation and Comparison

Multiple evaluation metrics were integrated to quantify predictive capacity, including the area under the ROC curve (AUC): for quantifying the model’s capacity to differentiate patients with anxiety from nonanxiety counterparts. Accuracy: represented the total share of samples with accurate classification. Sensitivity and specificity quantified the model’s recognition accuracy for anxiety patients and normal participants separately, maintaining stable predictive effects on positive and negative samples. Based on a systematic comparison of these indicators, the model achieving optimal predictive effects within the testing cohort was designated as the ultimate prediction instrument. Furthermore, variable importance analysis of the optimal model was conducted to identify key predictive factors influencing anxiety status, providing targeted insights for clinical intervention.

### 3.9. Analysis Tools

All analyses were performed using R (Version 4.3.3), primarily employing the packages randomForest, e1071, nnet, caret, and pROC. The statistical significance threshold was set at *α* = 0.05.

### 3.10. Comparison of Performance Metrics Among the Three Machine Learning Models

ROC curves of the three machine learning models for anxiety status prediction are shown in Figure 1.

**Figure 2:**
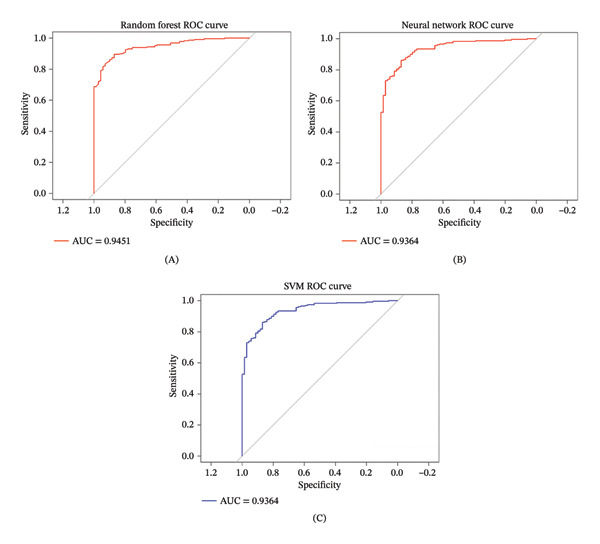
ROC curves of the three machine learning models for anxiety status prediction.

A total of 1042 potential participants were initially enrolled. After excluding 42 participants due to excessive missing data on questionnaires, the final analytical dataset contained 1000 qualified subjects. All samples underwent random stratification: 700 cases (70%) formed the training cohort, with the remaining 300 samples (30%) reserved as the testing subset (Figure 2).

ROC curves for comparison were generated for three predictive algorithms—RF, SVM, and ANN—assessed on the reserved testing subset accounting for 30% of all samples (*n* = 300). Following SMOTE balancing, model training was implemented uniformly using the 700‐sample training cohort.

A: ROC curve of the RF model, with an AUC of 0.945, approaching 1.0, indicating excellent discriminative ability. The curve achieves elevated sensitivity alongside minimal false alarm rate, reflecting outstanding discriminatory power for distinguishing anxious and nonanxious subjects. B: ROC curve of the ANN model, with an AUC of 0.8916. The curve shape indicates good discriminative ability, though slightly inferior to the RF and SVM models, suggesting a weaker capacity to distinguish between the two groups. C: ROC curve of the SVM model, with an AUC of 0.9364. DeLong’s test for paired ROC curves was adopted to compare intermodel AUC disparities and verify statistical significance. The DeLong test showed that the AUC of the SVM model (0.9364) exhibited statistically superior values relative to the ANN model (0.8916, *p* = 0.012), its value showed no statistical discrepancy versus RF (0.9451, *p* = 0.214). These results indicate that while SVM and RF have comparable discriminative ability, both significantly outperform ANN in this dataset.

Note: AUC = area under the receiver operating characteristic curve; ROC = receiver operating characteristic.

### 3.11. SVM Analysis Method and Results

**Figure 3:**
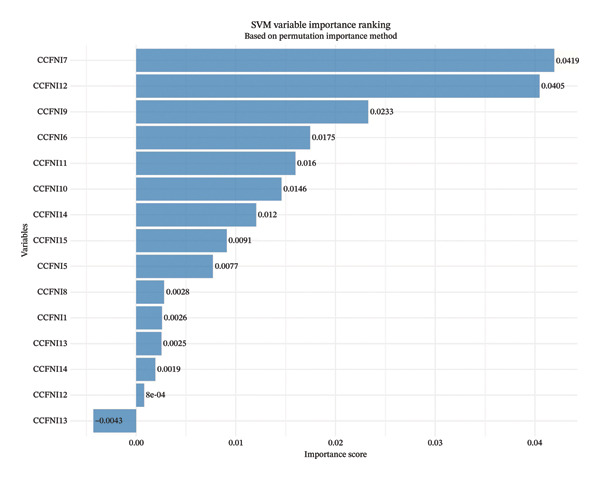
Variable importance ranking of the support vector machine model.

**Figure 4:**
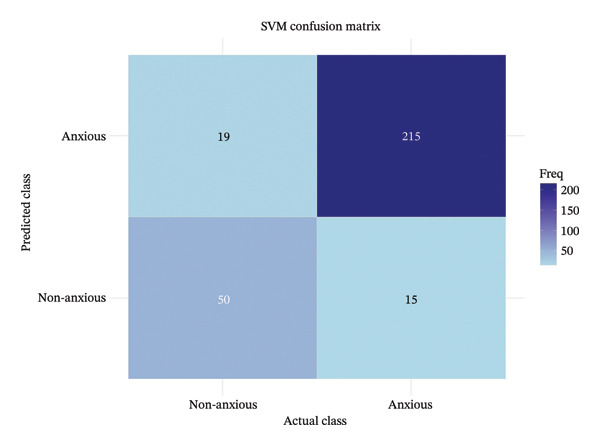
Confusion matrix of the support vector machine model on the test set.

**Figure 5:**
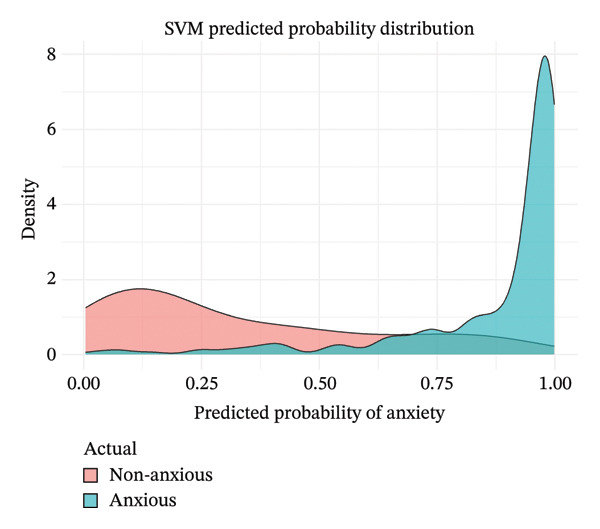
Prediction probability distribution of the support vector machine model.

The core goal of SVM is solving for an optimal hyperplane to widen the separation interval of the two sample categories. For the linearly nonseparable anxiety data used in this study, the RBF kernel maps raw predictors onto a high‐dimensional feature domain allowing a linear hyperplane to be identified for classification. The kernel coefficient (*γ*) determines the influence range of each sample on the decision boundary—larger values produce more localized effects and a more complex model. The penalty parameter (C) controls the trade‐off between classification accuracy (penalizing misclassified samples) and model generalization (margin width). Grid search coupled with 10‐fold cross‐validation screened ideal parameter combinations inside the preset parameter range (: [0.1, 1, 10, 100]: [0.001, 0.01, 0.1, 1]). To generate calibrated probability outputs, Platt scaling was employed. This method fits the raw SVM decision scores to a sigmoid function, transforming them into probabilistic class estimates (Figures 3, 4, and 5). The analysis results of RF and ANN are shown in Supporting S1–S8 of the Supporting Information.

Feature importance for SVM: Because classical SVM models with RBF kernels do not directly provide interpretative feature coefficients, we employed permutation feature importance to rank variables. This approach quantifies declines in AUC following random permutation of individual predictors with larger drops indicating greater importance. The importance ranking was consistent across 100 permutations.

Variable importance ranking is derived from the SVM model using permutation feature importance. Because classical SVM models with RBF kernels do not directly provide interpretable feature coefficients, permutation importance was employed: each feature was randomly shuffled 100 times, and the drop in model performance (AUC) was measured. Larger drops indicate greater importance. Predictors are sorted vertically from highest to lowest importance values; the horizontal coordinate reflects average AUC loss postfeature permutation, normalized to a maximum value of 100.

The top four CCFNI items predicting anxiety status were CCFNI_4, CCFNI_7, CCFNI_9, and CCFNI_12. These items span the domains of information acquisition, prognostic communication, emotional reassurance, and care coordination. Their high importance rankings were consistent across all three machine learning models (SVM, RF, and ANN).

CCFNI = Critical Care Family Needs Inventory; AUC = area under the ROC curve.

This figure displays the classification outcomes of the SVM model on the test dataset. The model correctly predicted 50 nonanxiety cases (true negatives) and 215 anxiety cases (true positives), with 4 false positives (nonanxiety predicted as anxiety) and 81 false negatives (anxiety predicted as nonanxiety). These results confirm the high accuracy and strong practical applicability of the SVM model, demonstrating its reliability in differentiating between anxiety and nonanxiety groups among family members of Neurosurgical ICU patients in clinical settings.

This figure depicts the probability distributions of predictions generated by the SVM model. The probability distributions of the nonanxiety and anxiety groups exhibited minimal overlap, with a clearer separation than that achieved by the RF and ANN models. This finding indicates that the SVM model optimized its discrimination threshold more effectively, supporting accurate anxiety risk stratification for relatives of NICU admitted patients.

## 4. Discussion

Distress experienced by relatives of critically ill hospitalized patients has drawn rising attention within intensive care research. Johnson et al. reported that the prevalence of anxiety symptoms in ICU patients’ family members ranges from 2% to 80%, with several large‐scale studies indicating that approximately 66% of the family members develop clinically significant anxiety during the hospitalization period [15]. Similarly, Omoregie et al. found that up to 90% of the family members experience pronounced depressive symptoms attributable to factors such as including high treatment costs, frequent patient deaths in the ICU, and limited involvement in patient care [16]. De Miranda et al. demonstrated that among the 72.2% of family members who experienced anxiety symptoms at ICU discharge, 29.8% developed post‐traumatic stress disorder–related symptoms within 90 days, a rate significantly exceeding than the rate observed in the nonanxious group [17]. These findings indicate that the prevalence of anxiety among ICU patients’ family members is far greater than traditionally recognized. Consistent with our conceptual framework, the results confirm that specific unmet needs measured by the CCFNI—particularly those related to honest information, prognostic expectations, and reassurance—are strong predictors of anxiety, supporting the use of anxiety as a surrogate outcome for identifying clinically significant unmet family needs. Given the severity and unpredictability of neurological conditions in Neurosurgical ICU patients, as well as the high uncertainty of outcomes, rates of anxious mood in their kin notably exceed those of relatives under general intensive care treatment only 12 participants (1.2%) exhibited no anxiety symptoms, while 768 (76.8%) experienced moderate to severe anxiety. Bugri et al. also reported that family members of patients with TBI not only exhibit a high incidence of anxiety but also encounter interpersonal strain, role‐related challenges, and stress‐associated health issues, often continuing to experience persistent psychological distress even after the patient’s discharge [18].

The theoretical premise that CCFNI items reflect latent unmet family demands is grounded in both scale development literature and established family stress theory. The original construction of the CCFNI explicitly incorporated items to capture unspoken, unaddressed concerns of ICU relatives beyond straightforward informational requests. From the perspective of the family stress process model, continuous unmet care needs constitute persistent environmental stressors that correlate with sustained emotional discomfort. The present study leverages this established theoretical framework to examine associations between multidimensional demand deficits and anxiety symptoms, rather than drawing unsubstantiated speculative connections between CCFNI items and latent needs.

The newly identified specialized triggers of anxiety represent a breakthrough beyond the traditional ICU research framework. Acaroğlu emphasized that, in addition to conventional stressors, Neurosurgical ICU‐specific factors—such as early neurological deterioration signals, the high intensity of invasive procedures, and uncertainty in prognostic evaluation—are central contributors to the compounded anxiety experienced by family members [19]. Notably, when patients develop delirium, family members’ anxiety levels can increase to 2.1 times their baseline [20]. Furthermore, the decision‐making burden associated with determining surgical timing and weighing life‐sustaining treatment options exerts a substantially greater psychological impact on spouses than on other relatives.

Currently, precision nursing interventions are shifting toward a model of medical–family collaboration. Muehlschlegel et al. demonstrated that various nursing intervention strategies can effectively reduce anxiety scores among family members in the Neurosurgical ICU [21]. Likewise, Hwang reported that a multidisciplinary communication team—including neurologists, specialized nurses, and psychologists—significantly improves family members’ understanding of the patient’s condition, thereby alleviating anxiety [22]. Although these studies have achieved notable progress, significant perceptual gaps persist between medical staff and family members concerning multiple various influencing factors. This discrepancy arises from the fact that the familial role burden extends beyond decision‐making to include responsibilities such as information transmission and family coordination, which further amplify anxiety levels [23].

Variable importance analysis revealed that the top four CCFNI items predicting the anxiety status were CCFNI_4, CCFNI_7, CCFNI_9, and CCFNI_12. These items span the domains of information acquisition, prognostic communication, emotional reassurance, and care coordination. Zhao et al. constructed a conceptual framework for translating predictive molecular and clinical indicators into practical clinical intervention strategies, which can be referenced when converting our screened core family demand indicators into targeted nursing measures [23]. The identification of these four CCFNI items as the top predictors of anxiety has direct implications for nursing management. First, nursing administrators should prioritize structured communication protocols ensuring honest and timely information delivery. Second, regular prognostic discussions involving neurologists, specialized nurses, and family members should be scheduled to manage expectations. Third, explicit reassurance strategies such as daily briefings on the quality of care should be integrated into standard Neurosurgical ICU workflows. Fourth, automated notification systems for condition changes can address CCFNI_12 without increasing nursing workload. By targeting these four core needs, nursing managers can allocate resources more efficiently and standardize psychoanalytical support procedures.

Interventions targeting unmet family demands may help relieve anxiety symptoms among ICU relatives, rather than only relying on superficial emotional reassurance. Blok et al. noted that high anxiety levels among family members tend to correlate with cumulative unmet hidden needs—such as unfamiliarity with medical procedures, vague prognostic information, and limited participation in clinical decision‐making [24]. Because these needs are frequently concealed, they are easily overlooked in routine nursing practice, leading to the progressive intensification of anxiety symptoms. Moreover, the deeper needs of ICU family members exhibit strong situational specificity and significant individual variation. Hwang et al. demonstrated the primary needs of family members differ according to patient’s condition [25]; for example, relatives of patients with acute traumatic injuries place greater emphasis on the timeliness and efficacy of treatment plans, whereas those of patients with chronic critical illnesses are more concerned with obtaining clear and comprehensive assessments of long‐term prognosis. Family role also influences the pattern of needs: spouses express an approximately 1.8 times stronger demand for participation in decision‐making compared with other relatives, while elderly family members show an unmet need rate as high as 59% for simplified explanations of medical terminology. The complexity and heterogeneity of these needs indicate that accurate responses cannot rely solely on conventional nursing judgment [26, 27].

Perceptual gaps between medical staff and family members are significantly correlated with elevated anxiety symptoms among relatives [28–30]. Comparative studies have shown that ICU medical staff score approximately 42% lower than family members in assessing the importance of emotional support and real‐time condition feedback. This perceptual discrepancy leads to a divergence between implemented interventions and the actual needs of family members. Healthcare providers often concentrate on conveying information about treatment progress while neglecting family members’ concerns regarding aspects such as the patient’s pain perception and psychological changes among other aspects. Unmet demands are observed to be correlated with more severe anxiety symptoms among family members. This observation supports the value of this study, which aims to predict family anxiety and screen core CCFNI items linked to unmet demands.

In constructing a demand prediction model for family members of ICU patients based on the CCFNI, the ANN, RF, and SVM models demonstrated clear advantages over traditional statistical methods. The primary limitations of conventional statistical approaches lie in their reliance on linear assumptions and oversimplified modeling structures. Classical methods such as logistic regression require the a priori specification of linear relationships among variables; however, the needs of ICU family members are influenced by multiple interacting dimensions—including patient condition severity, family roles, and communication quality—whose nonlinear interdependencies are difficult to capture using these approaches. Existing literature exploring care demands of aging ICU patients’ kin indicated that ignoring interaction terms among variables may trigger substantial forecasting biases. For instance, the error rate for predicting complex needs such as “the desire for prognostic information” reached as high as 37% in traditional statistical models, substantially higher than that of machine learning algorithms. Moreover, traditional models exhibit limited capacity for handling high‐dimensional data; when the number of CCFNI‐derived features exceeds 20, multidisciplinary frequently arises, thereby undermining model stability and predictive reliability [31, 32].

The systematic comparison conducted in this study highlights the distinctive advantages and applicable scenarios of the three machine learning algorithms in predicting anxiety states among family members of ICU patients. Among them, the SVM achieved the best overall balance, demonstrating the highest accuracy (0.8863) and F1 score (0.7463), along with strong sensitivity (0.7264) and specificity (0.9348). These results indicate that the SVM model effectively balances accurate identification of anxious individuals (high sensitivity) with minimization of false positives for nonanxious individuals (high specificity). Such balance is critical for clinical screening tools, as both missed diagnoses (low sensitivity) and misdiagnoses (low specificity) can lead to significant emotional and medical consequences. The high AUC value (0.9364) further confirms the model’s strong discriminating ability. In comparison, the RF model exhibited exceptionally high specificity (0.9565) and the highest AUC value (0.9451), suggesting excellent ability to correctly classify nonanxious individuals and robust ranking performance. However, its relatively low sensitivity (0.5362) represents a notable limitation, as it implies that approximately 46% of the anxious individuals could be missed—a rate that is unacceptable for early psychological screening. Therefore, although RF provides stable results and clear interpretability in variable importance analysis (highlighting CCFNI_4, CCFNI_7, and CCFNI_9 as core predictive factors), its applicability as a standalone screening model is constrained by its high rate of missed detection. The ANN demonstrated the weakest overall performance among the three models, particularly in precision (0.6184) and AUC (0.8916). Such a phenomenon could be explained by the restricted scale of enrolled subjects, which restricts the ANN’s ability to fully optimize its parameters and realize its learning potential. Although the model’s sensitivity (0.6812) was higher than that of the RF, its overall predictive performance remains insufficient for use as a primary clinical tool.

In summary, on the basis of the present findings, the SVM model is recommended as the preferred approach for anxiety prediction. The rationale is as follows: (1) Optimal clinical applicability: its high and well‐balanced sensitivity and specificity fulfill the essential requirements of screening tools; (2) Strong nonlinear modeling capability: the complex associations between variables and anxiety states are effectively captured through the RBF kernel; and (3) Robust performance against overfilling: model complexity is efficiently controlled through appropriate tuning of the penalty parameter (C) and kernel coefficient (*γ*).

### 4.1. Limitations

This study has certain limitations. Foremost among these is the absence of an external validation datasets. While we performed an internal train–test split (7:3), the model’s performance may be overestimated, and its generalizability to other Neurosurgical ICU populations remains unknown. Future studies should validate the SVM model on independent, multicenter cohorts from diverse geographic regions and healthcare systems. The sample size may limit the generalization of the SVM model, and its full predictive potential has yet to be explored. Additionally, furthermore, the study did not integrate the high‐precision classification capability of the SVM with the interchangeability of variable importance offered by the RF, which could limit the development of a more accurate and transparent clinical decision support system.

Single‐center data collection restricts broad extrapolation of the SVM classifier to neurosurgical ICUs differing in case composition, institutional atmosphere, and resource allocation.

## 5. Conclusions

This study systematically examined anxiety among family members of ICU patients and revealed that its prevalence substantially exceeds traditional expectations. Family members of Neurosurgical ICU patients show elevated anxiety levels that correlate with multiple overlapping factors including Neurosurgical ICU–specific stressors and decision‐making burdens, which supports the value of targeted intervention research. Unmet fundamental family demands are closely associated with higher anxiety severity, and perceptual gaps between medical staff and relatives show additional correlations with aggravated psychological distress. Restricted by linear assumptions and weak performance with high‐dimensional data, traditional statistical models have limited capacity to predict family anxiety and screen core CCFNI items related to psychological distress. In contrast, machine learning algorithms exhibit clear advantages, SVM outperformed alternative algorithms when balancing detection accuracy and true negative screening capacity. Although the RF model achieved a higher AUC, its low sensitivity precludes its use as a standalone screening tool. Based on CCFNI demand indicators, the SVM classifier serves as a dependable clinical screening instrument for anxiety risk prediction among neurosurgical ICU relatives and to pinpoint the key unmet family needs driving their psychological distress, thereby enabling precise identification of their underlying needs and fostering effective medical–family collaboration. This approach not only helps alleviate anxiety among family members of ICU patients but also offers a new direction for advancing humanistic nursing in critical care medicine.

**Nomenclature**
NICU: Neurosurgical intensive care unit
HADS: Hospital Anxiety and Depression Scale
CCFNI: Critical Care Family Needs Inventory
sTBI: Severe traumatic brain injury
GCS: Glasgow Coma Scale
CT: Computed tomography
MRI: Magnetic resonance imaging

## Funding

This research was supported by Yijishan Hospital Level Service Management Innovation Project (cx2024008), School Level Key Humanities and Social Sciences Project of Wannan Medical University (wk2024szd01), and Teaching Reform Research Project of Wannan Medical University (2025jyxm011).

## Ethics Statement

All methods were carried out in accordance with relevant guidelines and regulations. The study was carried out respecting the Declaration of Helsinki in its current version. The study was approved by the Medical Ethics Committee of the First Affiliated Hospital of Wannan Medical University (no. 2018043), and written informed consent was obtained from all the participants.

## Consent

All patients included in this study had signed informed consent.

## Conflicts of Interest

The authors declare no conflicts of interest.

## Supporting Information

Additional supporting information can be found online in the Supporting Information section.

## Supporting information


**Supporting Information 1** The Supporting Information provide the complete analytical results of the Random Forest (RF) and Artificial Neural Network (ANN) anxiety prediction models. Data were derived from the Critical Care Family Needs Inventory (CCFNI) cohort of 1000 NICU patient family members. While the SVM model (identified as optimal) is detailed in the main manuscript, the full findings of the RF and ANN models are presented here as Supporting Information.

**Supporting Information 2** STROBE_checklist_v4_combined.

## Data Availability

The data that support the findings of this study are available from the corresponding author upon reasonable request. Due to institutional ethics restrictions (patient privacy protection), the data are not publicly available.
