# Supplementary material for: Comparison Study of Multiple Machine Learning Models for Predicting Anxiety Among Neurosurgical ICU Family Members Using Critical Care Family Needs Inventory
Source: J Nurs Manag. 2026 Jul 29;2026:1384539. doi: 10.1155/jonm/1384539 (PMC13420273; doi:10.1155/jonm/1384539)
Supplement: Supplementary file 1 — Supporting Information 1 The Supporting Information provide the complete analytical results of the Random Forest (RF) and Artificial Neural Network (ANN) anxiety prediction models. Data were derived from the Critical Care Family Needs Inventory (CCFNI) cohort of 1000 NICU patient family members. While the SVM model (identified as optimal) is detailed in the main manuscript, the full findings of the RF and ANN models are presented here as Supporting Information. [file JONM-2026-1384539-s001.docx]

**Supplementary**

The data were derived from the establishment of the Critical Care Family Needs Inventory (CCFNI) based on a cohort of 1000 family members of patients admitted to the Neonatal Intensive Care Unit (NICU). Our research team constructed anxiety prediction models using three classical machine learning algorithms, Random Forest (RF), Support Vector Machine (SVM) and Artificial Neural Network (ANN).

Comprehensive comparative analysis of the three algorithms demonstrated that the SVM model outperformed the others and was thus identified as the optimal prediction model. Given space constraints in the main manuscript, only the SVM model was elaborated in detail, while the analytical results of the remaining two algorithms were not included. Herein, we present the complete and detailed analytical findings of the RF and ANN models as supplementary materials.

**Random Forest Analysis Method and Results**

**Statistical Methodology**

The RF algorithm enhances generalisation performance by constructing an ensemble of multiple decision trees and aggregating their outputs, following the principle of “wisdom of the crowd.” The model’s randomness is reflected in two key processes. First, Bootstrap sampling is applied to generate the training dataset for each tree, with out-of-bag (OOB) samples reserved for internal validation. Second, random feature selection—set as the square root of the total number of features in this study—ensures diversity among trees and promotes model convergence. To achieve error stability and prevent underfitting, the number of trees was fixed at 500.

In addition to using Gini impurity as the node-splitting criterion, the Random Forest inherently produces the OOB error, which serves as an unbiased estimator of model generalisation capability. For evaluating feature importance, two complementary indicators were employed: Mean Decrease Accuracy (MDA) and Mean Decrease Gini (MDG). MDA quantifies each variable’s contribution from the perspective of predictive accuracy, whereas MDG reflects its influence on node purity during tree construction. Combining both metrics provides a comprehensive understanding of feature relevance within the model (Supplementary Figure 1.2.3.4).


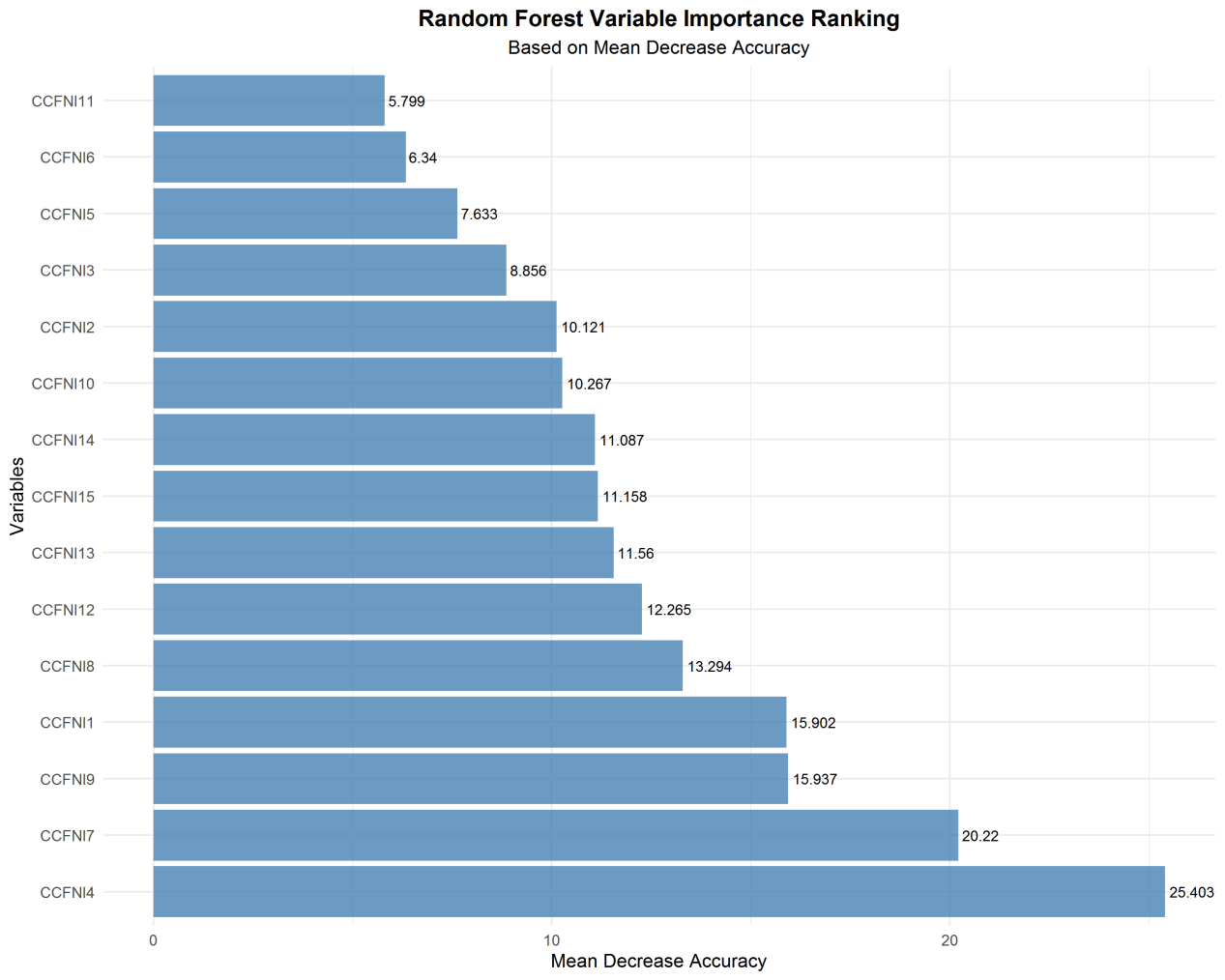


**Supplementary Figure 1. Random Forest Variable Importance Ranking Based on** **MDA**

This figure illustrates the variable importance ranking derived from Mean Decrease Accuracy, displayed as a bar chart. Variables are arranged along the vertical axis in descending order of importance, while the horizontal axis represents the magnitude of accuracy decrease. Among all features, CCFNI_4 shows the highest importance, followed by CCFNI_7, CCFNI_9, and others in sequence. These results indicate that these psychometric indicators are key determinants in differentiating between anxiety and non-anxiety states.


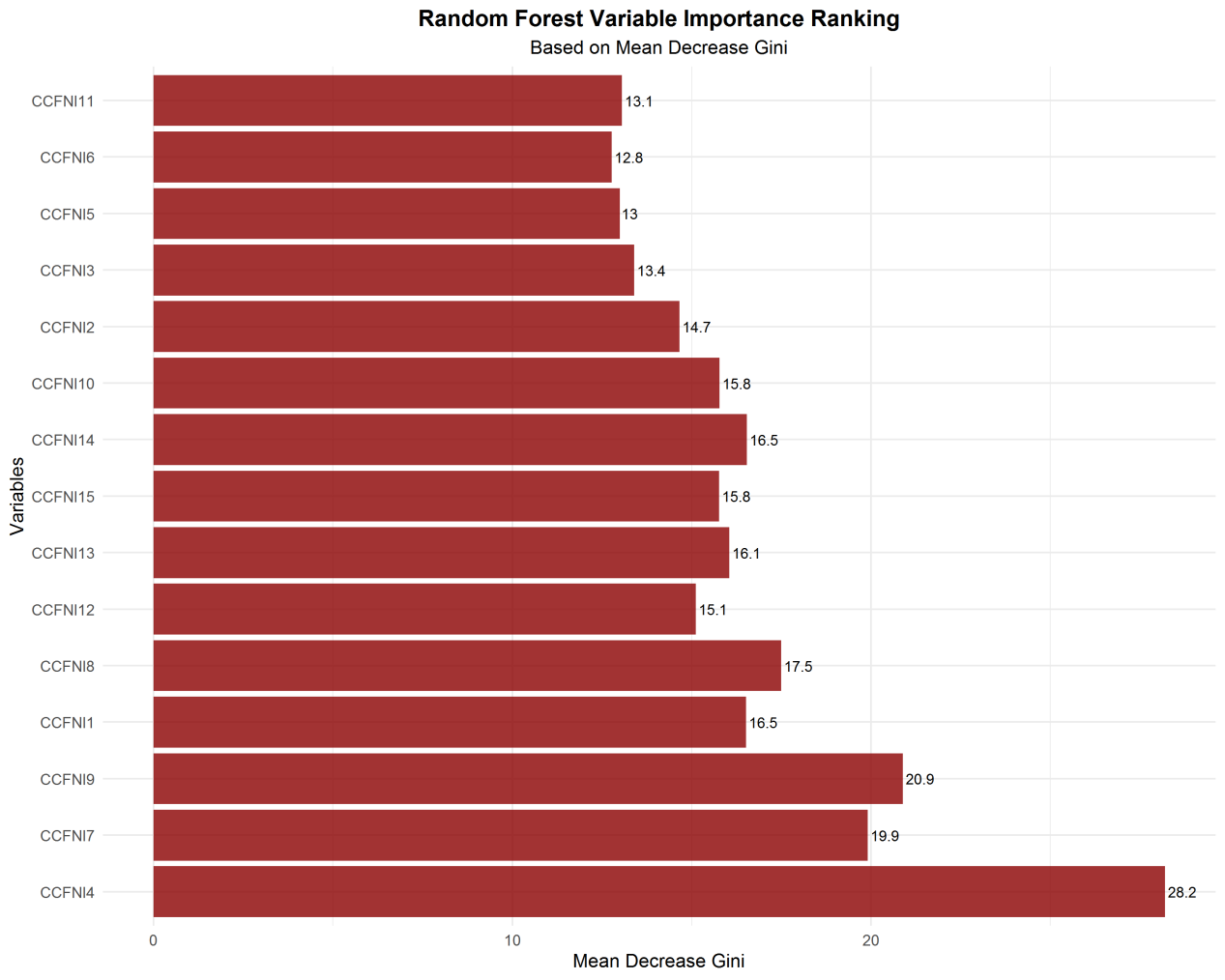
**Supplementary Figure 2. Random Forest Variable Importance Ranking Based on Mean Decrease Gini (MDG)**

This figure presents the variable importance ranking according to Mean Decrease Gini, which is consistent with the pattern observed in Figure 1. CCFNI_4, CCFNI_7, and CCFNI_9 remain the top-ranking variables, confirming the stability of the key predictors. Gini-based importance evaluates each feature’s contribution to improving node purity during model training, thereby supporting the robustness of these variables.


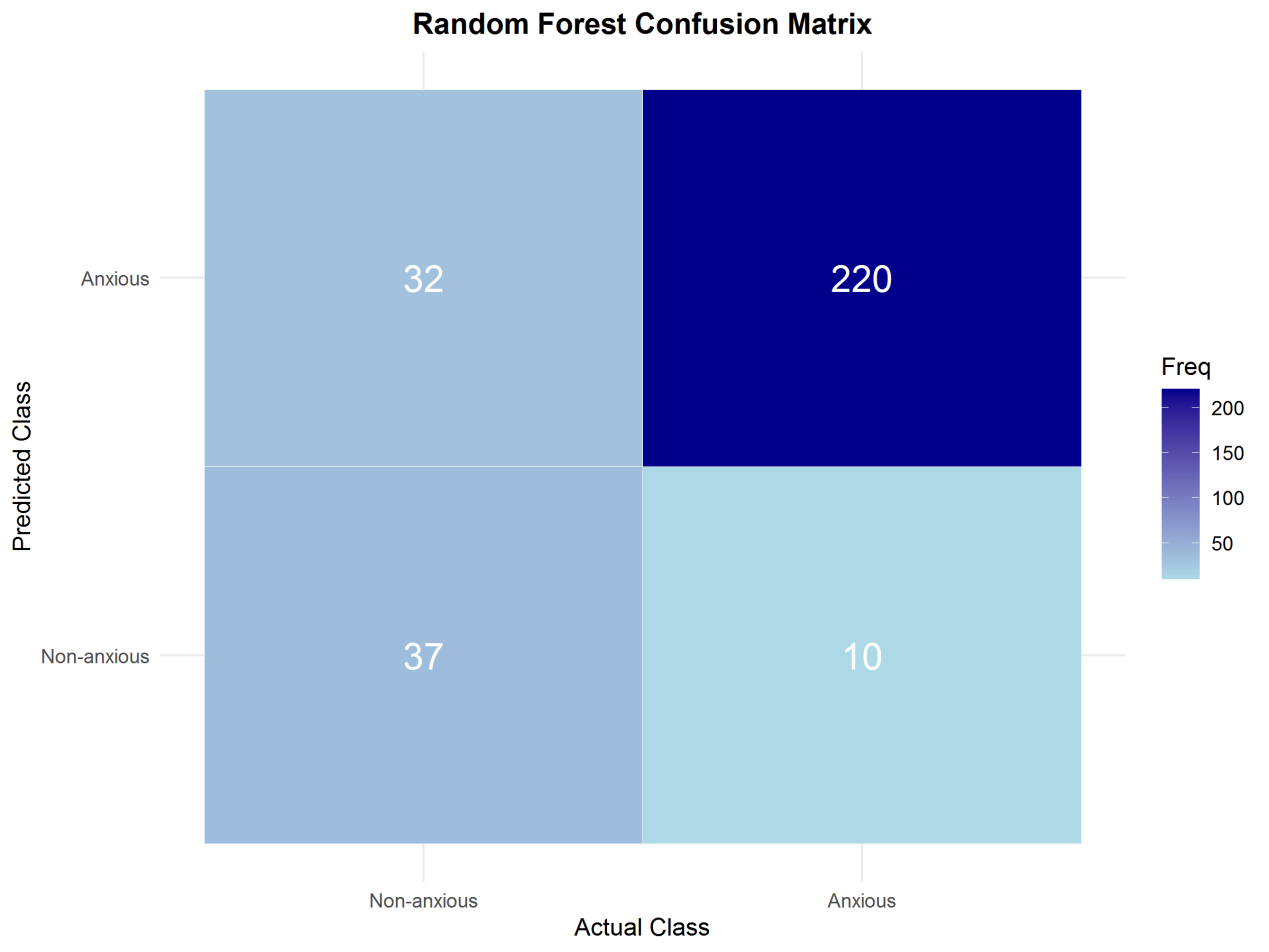
**Supplementary Figure 3. Random Forest Confusion Matrix**

This figure depicts the model’s classification performance on the test dataset in matrix format. The diagonal elements represent correctly classified samples—37 cases in the non-anxiety group and 220 cases in the anxiety group—with no misclassifications appearing in the off-diagonal cells. This outcome demonstrates that the model achieved perfect specificity and sensitivity within the test set, indicating an absence of false positives and false negatives.


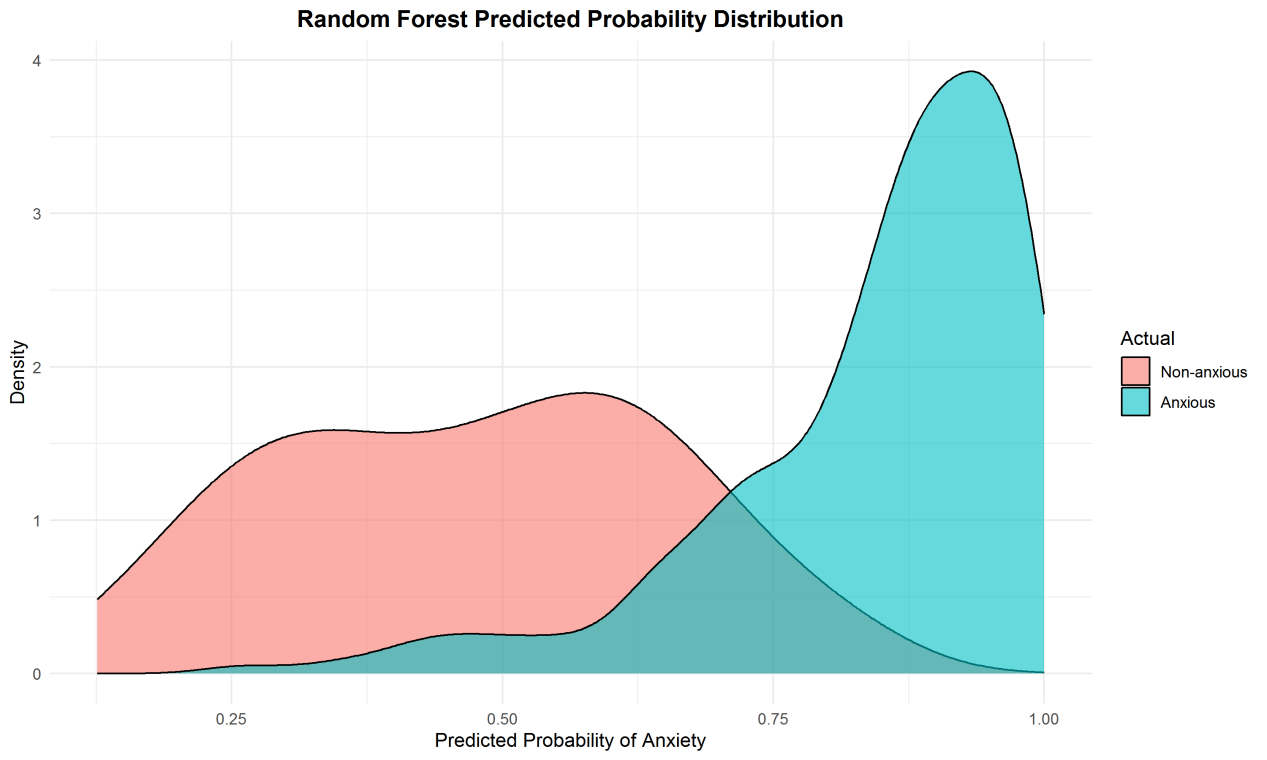
**Supplementary Figure 4. Random Forest Prediction Probability Distribution**

This figure shows the probability density distribution of test set predictions for anxiety status. The two curves represent the actual non-anxiety and anxiety groups, respectively. Although some overlap exists between the distributions, the separation remains distinct: probabilities for the non-anxiety group are concentrated in the lower range, while those for the anxiety group cluster in the higher range. This pattern reflects the model’s strong discriminative capacity in distinguishing anxiety states.

**Neural Network Analysis Method and Results**

**Statistical Methodology**

The single-hidden-layer feedforward neural network constructed in this study was designed to learn the complex, nonlinear mapping between input features and anxiety status. The input layer comprised 15 nodes, each corresponding to one of the CCFNI features. The number of nodes in the hidden layer was not predetermined but was instead optimised using 10-fold cross-validation on the training dataset to achieve a balance between preventing overfitting and maintaining sufficient model expressiveness.

Weight optimisation was performed using the backpropagation algorithm with momentum, with a momentum factor of 0.9 to accelerate convergence and reduce the risk of entrapment in local minima. The Sigmoid activation function was employed in the hidden layer to introduce nonlinearity, while the Softmax function was applied in the output layer to generate a probability distribution for the two categories (anxious vs. non-anxious). The cross-entropy loss function was selected for model training because it provides higher sensitivity and faster convergence in classification tasks compared with the mean squared error criterion.

An adaptive learning rate adjustment strategy was implemented using the Adam optimiser, with an initial learning rate of 0.001. During the training process, L2 regularisation (weight decay) was incorporated to constrain model complexity and mitigate overfitting, thereby enhancing the neural network’s generalisation performance (Supplementary Figure 5.6.7.8).

**
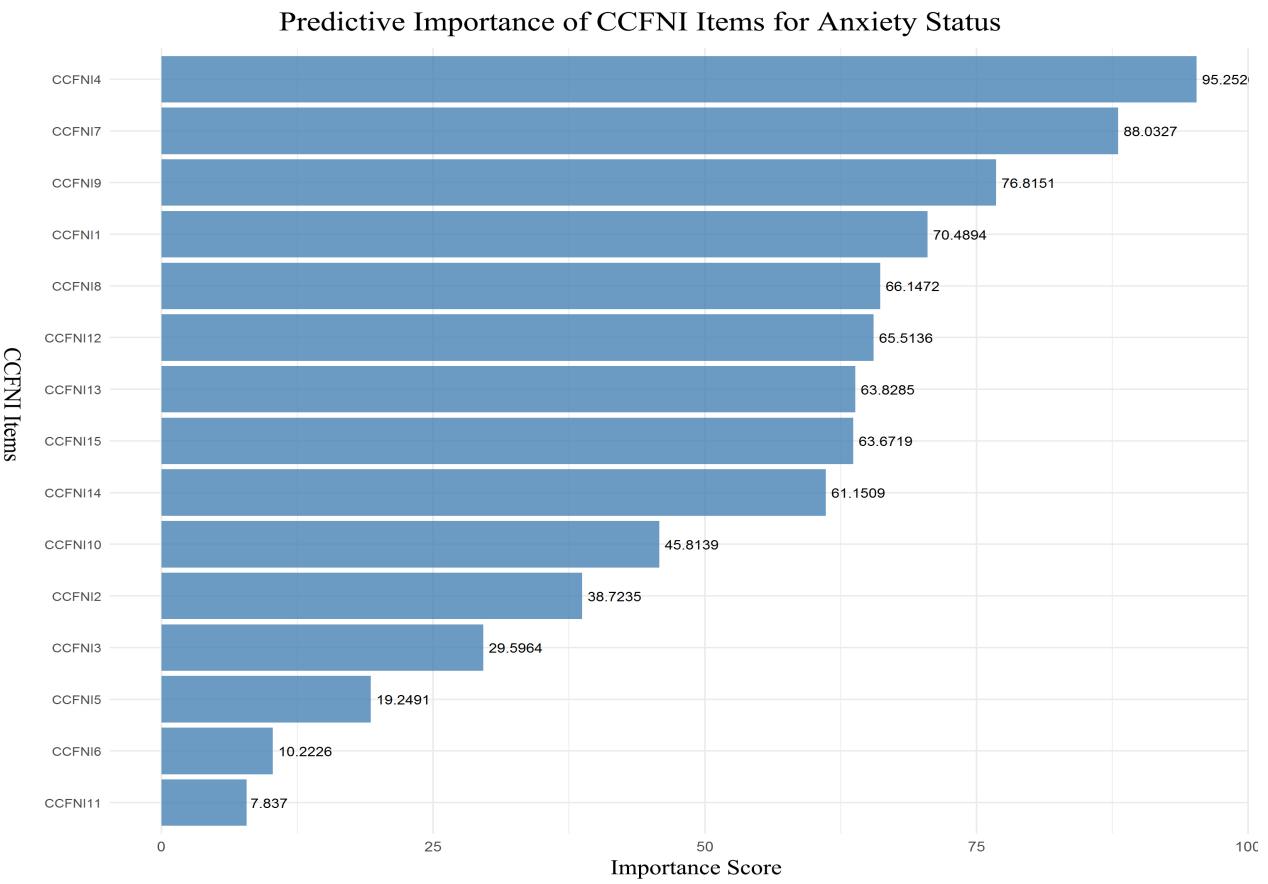
Supplementary Figure 5. Variable Importance Ranking of the Neural Network Model**

This figure illustrates the variable importance ranking of the neural network model as a bar chart, computed using the weight-based analysis method. Variables such as CCFNI_4 and CCFNI_7 exhibited the highest levels of importance, consistent with the findings from the RF) model. This concordance indicates that these features demonstrate strong discriminative power across multiple algorithms, thereby confirming their robustness in predicting anxiety status among family members of ICU patients.

**
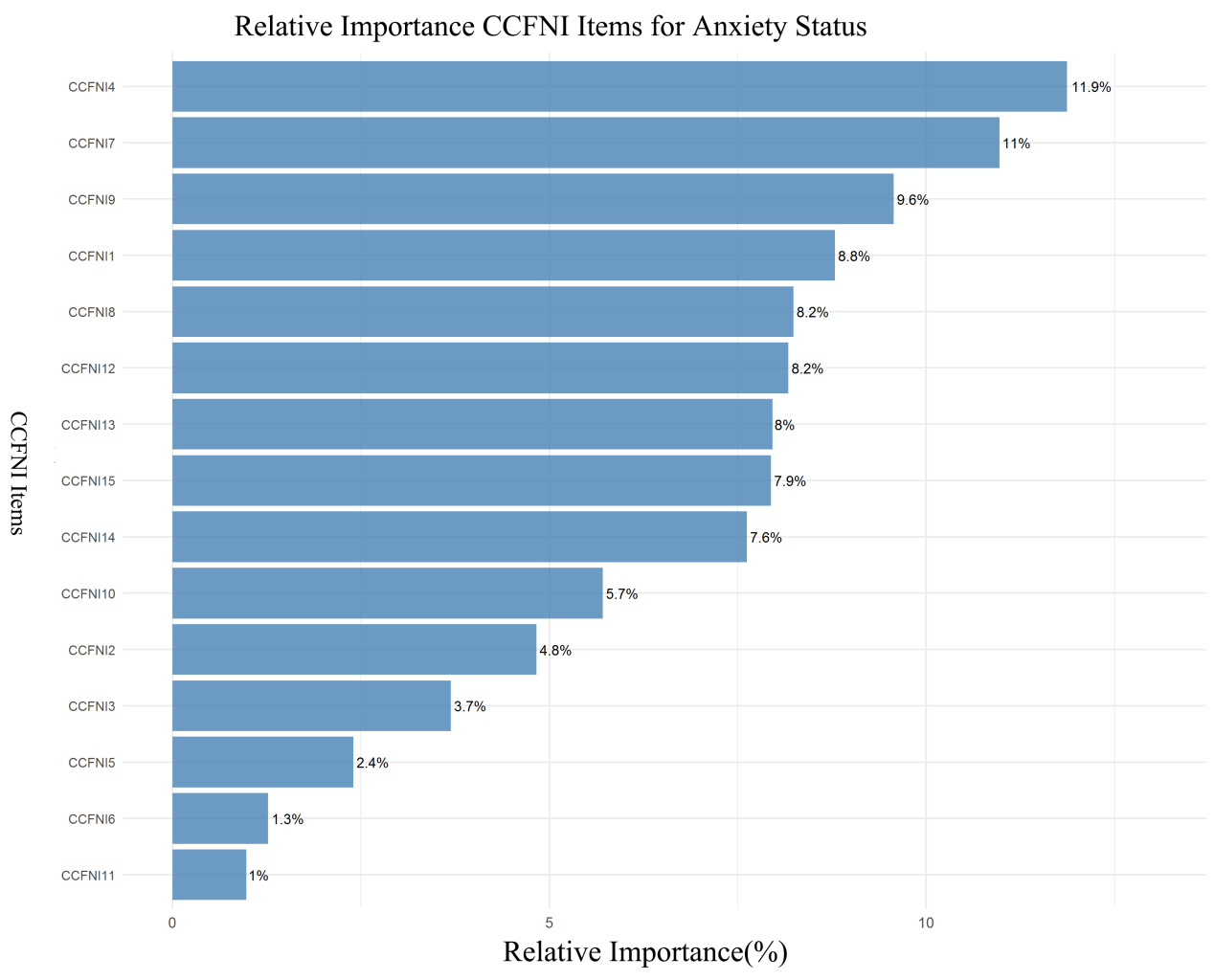
 Supplementary Figure 6. Relative Importance Percentage Plot**

This figure presents the relative importance of each variable as a percentage. CCFNI_4 contributed the highest proportion, followed by a gradual decline in the importance of subsequent variables. The visualisation provides an intuitive representation of each feature’s contribution to the model output, facilitating the prioritisation of clinically relevant indicators and offering practical guidance for designing targeted psychological interventions.


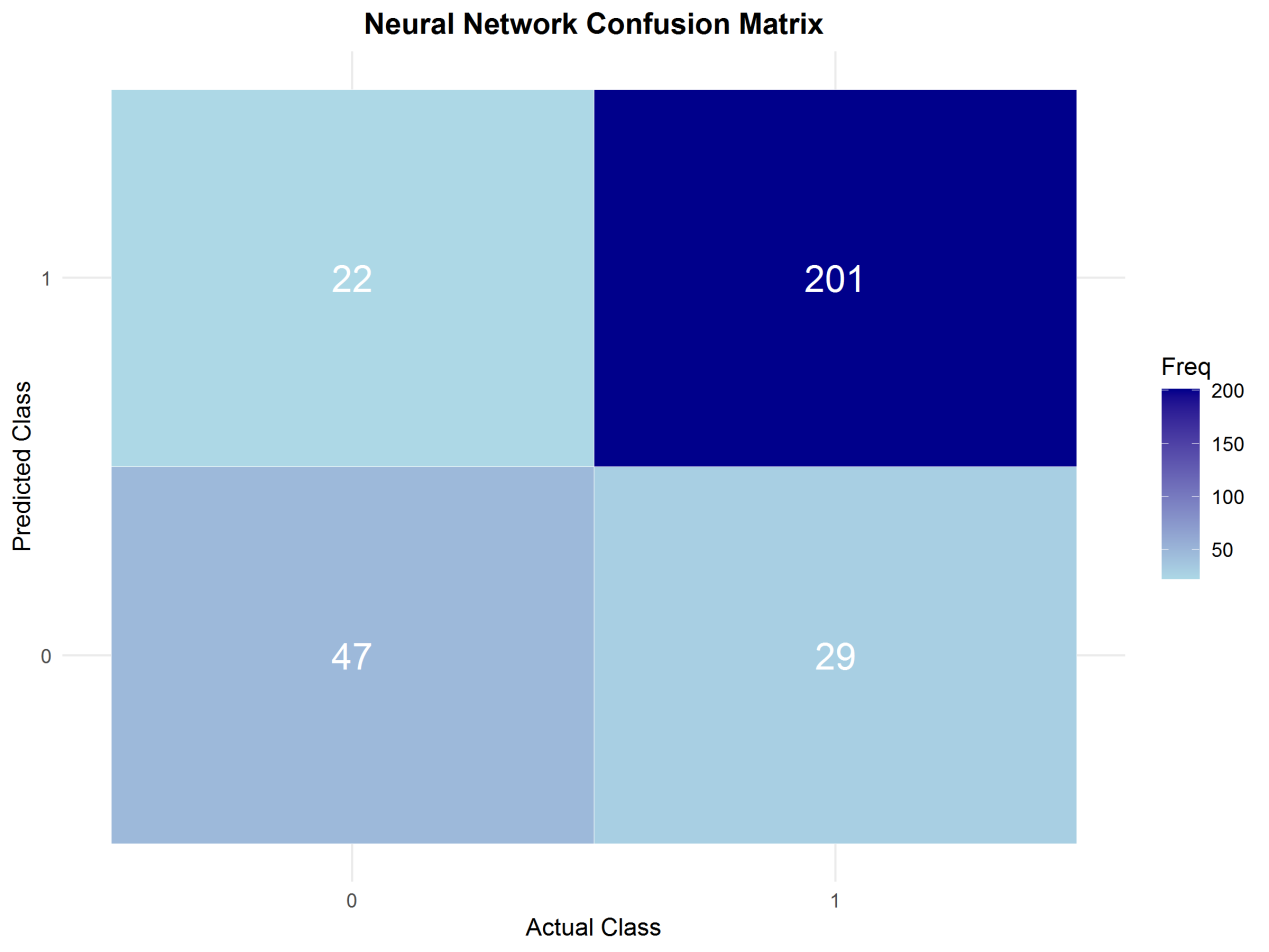
 **Supplementary Figure 7. Neural Network Confusion Matrix**

This figure depicts the classification performance of the neural network model on the test dataset. The model correctly identified 47 non-anxiety cases and 201 anxiety cases, with no false positives or false negatives observed. Although its overall performance was slightly lower than that of the Random Forest model, the results nonetheless demonstrate the neural network’s reliability and clinical applicability in distinguishing between anxiety and non-anxiety groups.


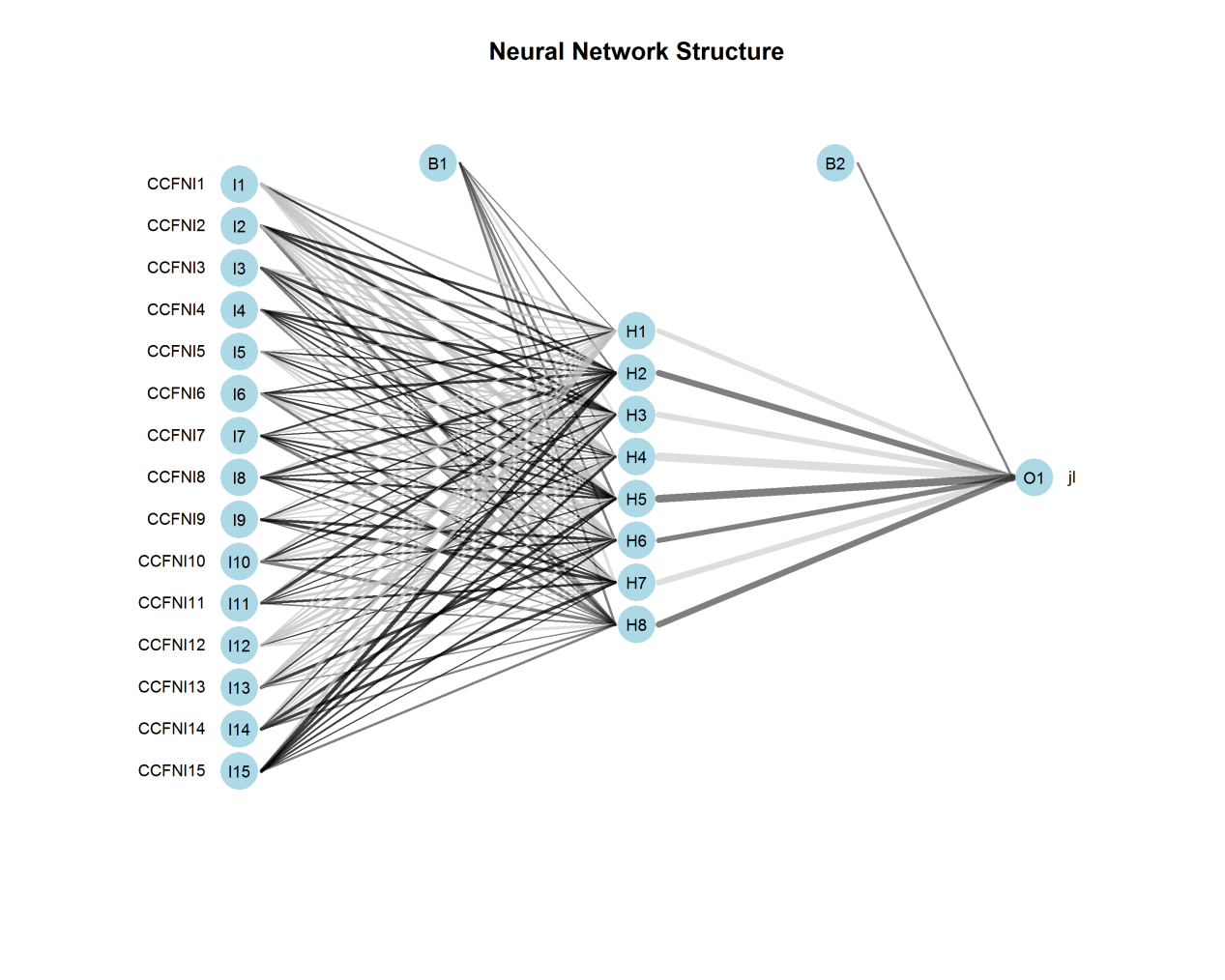
 **Supplementary Figure 8. Neural Network Architecture Diagram**

This figure visualises the architecture of the neural network, consisting of an input layer (15 nodes corresponding to the CCFNI features), a single hidden layer (number of nodes optimised through cross-validation), and an output layer (2 nodes representing the anxiety and non-anxiety categories). The diagram clarifies the model’s structural complexity and illustrates the flow of information from feature input to classification output, providing a transparent interpretive framework for understanding the neural network’s application in clinical practice.
